# GEMiCCL: mining genotype and expression data of cancer cell lines with elaborate visualization

**DOI:** 10.1093/database/bay041

**Published:** 2018-05-02

**Authors:** Inhae Jeong, Namhee Yu, Insu Jang, Yukyung Jun, Min-Seo Kim, Jinhyuk Choi, Byungwook Lee, Sanghyuk Lee

**Affiliations:** 1 Department of Bio-Information Science, Ewha Womans University, Seoul 03760, Republic of Korea; 2 Department of Life Science, Ewha Womans University, Seoul 03760, Republic of Korea; 3 Korean Research Institute of Bioscience and Biotechnology, Korean Bioinformation Center, Daejeon 34141, Republic of Korea; 4 Ewha Research Center for Systems Biology, Ewha Womans University, Seoul 03760, Republic of Korea

## Abstract

Cancer cell lines are essential components for biomedical research. However, proper choice of cell lines for experimental purposes is often difficult because genotype and/or expression data are missing or scattered in diverse resources. Here, we report Gene Expression and Mutations in Cancer Cell Lines (GEMiCCL), an online database of human cancer cell lines that provides genotype and expression information. We have collected mutation, gene expression and copy number variation (CNV) data from three representative databases on cell lines—Cancer Cell Line Encyclopedia , Catalogue of Somatic Mutations in Cancer and NCI60. In total, GEMiCCL includes 1406 cell lines from 185 cancer types and 29 tissues. Gene expression, mutation and CNV information are available for 1304, 1334 and 1365 cell lines, respectively. We removed batch effects due to different microarray platforms using the *ComBat* software and re-processed the entire gene expression and SNP chip data. Cell line names and clinical information were standardized using Cellosaurus from ExPASy. Our user interface supports cell line search, gene search, browsing for specific molecular characteristics and complex queries-based on Boolean logic rules. We also implemented many interactive features and user-friendly visualizations. Providing molecular characteristics and clinical information, we believe that GEMiCCL would be a valuable resource for biomedical research for functional or screening studies.

**Database URL**: GEMiCCL is available at https://www.kobic.kr/GEMICCL/

## Introduction

Cell lines are widely used in a variety of biomedical studies because of their unlimited supply, homogeneous characteristics, convenience and cost-effectiveness. Thousands of cell lines are commercially available from public and private resources such as ATCC and Sigma-Aldrich. They are routinely used for various purposes ranging from testing compound toxicity to production of eukaryotic proteins *in vitro*. Thus, cell lines are firmly established as an essential component of drug development.

Many immortalized cell lines originate from cancer cells. Both clinical and molecular characteristics are important in selecting cell lines suitable for a study design. Several attempts have been made to gather clinical information on frequently used cell lines (1, 2). Cellosaurus from ExPASy aims to be a knowledge resource on cell lines by providing extensive curated information that includes cell line names (recommended and synonyms), species, diseases, short tandem repeat, categories, publications and cross-references (3). Among the existing collection of databases with molecular information, CellLineNavigator provides mRNA expression information for a limited number of cell lines (4).

In line with the increasing importance of molecular characteristics of cell lines, several groups have performed large-scale genomic projects on cancer cell lines. Two notable examples are the Cancer Cell Line Encyclopedia (CCLE) project (5) by Broad-Novartis collaboration and the Sanger’s Catalogue of Somatic Mutations in Cancer (COSMIC) cell lines project (6), which independently yielded genetic and expression data for about 1000 cell lines. Although two thirds of their entries overlap, direct comparison is difficult because of their platform difference. Inefficient data visualization is another limiting factor for general users since CCLE was developed mostly for testing drug sensitivity and COSMIC was focused on somatic mutations. The canSAR database (7) maintains cell line information with mutation, copy number variation (CNV), expression data collected from COSMIC and NCI60, but no visualization or comparison is supported.

Prior knowledge of molecular and clinical characteristics including genetic abnormalities would allow more informed choice of cell lines in biological experiments and drug testing. Moreover, molecular information may be helpful in authentication and quality control of cell lines (8). Therefore, there is an immediate need for a comprehensive resource that provides molecular and clinical information in a unified manner. Gene Expression and Mutations in Cancer Cell Lines (GEMiCCL) integrated three representative genomic data resources to obtain gene expression, mutation and copy number alteration information. It covers the largest number of cancer cell lines to date, and our web interface supports diverse types of searches, browsing and comparison with intuitive visualization and interactive features.

## Materials and methods

### Cell line information

We standardized all cell line names as downloaded from the Cellosaurus (ftp://ftp.expasy.org/databases/cellosaurus) database (3). Disease names were selected from the MeSH terms, and tissue names were chosen using information provided by the COSMIC and CCLE databases.

### Mutation information

Since several databases did not provide raw sequencing data, we collected mutation information as provided from the original databases—COSMIC (CellLinesCodingMuts.vcf.gz file at http://cancer.sanger.ac.uk/cell_lines/download, Illumina exome sequencing), CCLE (CCLE_hybrid_capture1650_HGNC_info_2012.02.20.txt file from https://portals.broadinstitute.org/ccle/data, hybrid capture sequencing) and NCI60 (DNA: Sanger sequencing option at https://discover.nci.nih.gov/cellminer/loadDownload.do, Sanger and exome sequencing).

### Gene expression data analysis

Original microarray data were downloaded from COSMIC (https://www.ebi.ac.uk/arrayexpress/experiments/E-MTAB-3610, Affymetrix hg U219 array), CCLE (https://www.ncbi.nlm.nih.gov/geo/query/acc.cgi? acc=GSE36133, Affymetrix hg U133 plus 2.0 array) and NCI60 (https://www.ncbi.nlm.nih.gov/geo/query/acc.cgi? acc=GSE32474, Affymetrix hg U133 plus 2.0 array). We used the *Affy* R package (9) for RMA normalization and the hg19 genome for gene annotation. We further removed batch effects using the *ComBat* software (10). To compare gene expression values for the same cell line among three data sources, we calculated percentile values of gene expression for visualization.

### CNV data analysis

CNV values were obtained from SNP chip data for all three resources. We downloaded raw *CEL* files from the CCLE (https://www.ncbi.nlm.nih.gov/geo/query/acc.cgi? acc=GSE36138, SNP array 6.0) and NCI60 (https://www.ncbi.nlm.nih.gov/geo/query/acc.cgi? acc=GSE32264, Affymetrix GeneChip Human mapping 500 K array set) websites. COSMIC only provided processed results; thus, we downloaded the final results from the website (cell_lines_copy_number.csv file at http://cancer.sanger.ac.uk/cell_lines/download). Raw *CEL* files were processed using the *PennCNV-Affy* package (11) to obtain LRR (log R ratio) and BAF (B allele frequency) values. Then the CBS algorithm (12) in the *DNAcopy* R package was used for segmentation. Again, hg19 genome was used for gene annotation. To detect copy number aberrations, we sorted out the copy number values of all genes and identified two inflection points after LOESS curve fitting. Then, genes in the bottom and top 50 percentile beyond the inflection points were designated as copy number deletions and amplifications, respectively.

## Results

### System overview

**Figure 1.**
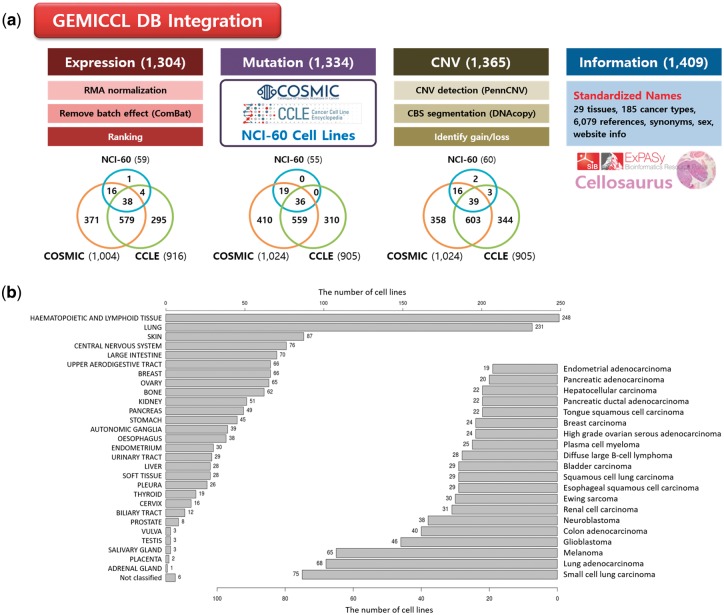
Overview of GEMiCCL database. (**a**) Data types and statistics according to source databases. Numbers in the parenthesis and the Venn diagram indicate the number of cell lines with relevant information. Gene expression and copy number data were reprocessed with our own pipelines as summarized. (**b**) Distribution of cell lines across 29 tissue types and top 20 cancer types.

GEMiCCL integrated three genomic resources for cell lines—COSMIC, CCLE, and NCI60. Figure 1 shows data types and statistics for each source database. Cell line names, tissues, diseases and cancer types were integrated via Cellosaurus. Overall, GEMiCCL covers gene expression, mutation and copy number data for 1304, 1334 and 1365 cell lines, respectively. The Venn diagrams show the number of cell lines with the corresponding data type from each resource, where COSMIC and CCLE are the two dominant contributors. The distribution of cell lines across tissues and cancer types is shown in Figure 1b, where cell lines from hematopoietic and lung cancers were most frequent. COSMIC and CCLE were the largest contributors, with almost half of the cell line entries having independent duplicate information from multiple resources.

One of the important advantages of GEMiCCL is that it allows users to compare molecular characteristics from different resources, which is only possible after removing batch effects and reprocessing raw data. In addition, whereas the original resources provide raw data (numbers) in a tabular format, our web interface supports elaborate searches/browsing with molecular output characteristics visualized in intuitive graphics. For example, smart searches like ‘melanoma cell lines with BRAF mutation’ or ‘lung cancer cell lines with high EGFR expression’ can be easily achieved by Boolean logic queries.

### Comparison of different DB sources

Each resource has varying platforms (e.g. microarray versions) and analysis methods. This leads to source-dependent batch effects, making direct comparison across different resources almost impossible. Whenever possible, we analyzed the raw data ourselves using our own pipelines and applied a statistical method to remove batch effects as indicated in the Materials and methods section.

**Figure 2.**
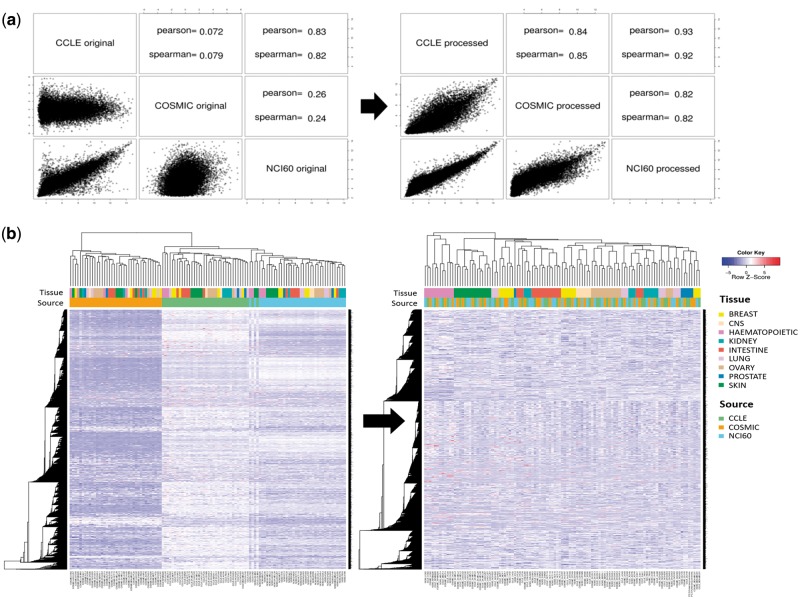
Batch effect in gene expression data. (**a**) Correlation plots and coefficients for gene expression data of A-549 cell line from three different resources. Left and right figures were obtained before and after removing batch effect with *ComBat* program. (**b**) Hierarchical clustering of gene expression data for 38 cell lines before and after removing batch effect. Pearson correlation coefficient and complete linkage were used in clustering.

Gene expression microarray data were available from all three resources. They were processed using a standard protocol for Affymetrix chips (i.e. *Affy* R package). Figure 2a shows the correlation of gene expression values for the A-549 cell line among the three resources. Reasonable agreement was obtained only after removing batch effects with the *ComBat* program. Note that CCLE and NCI60 data showed higher correlation probably because they were obtained from the same microarray version (hg U133 plus 2.0). Clustering of cell lines based on gene expression data yielded the same conclusion. As shown in Figure 2b, cell lines were grouped by source database before removing batch effects, whereas they were grouped by tissue type after applying the *ComBat* program. Thus, direct comparison was possible only after removing batch effects.

**Figure 3.**
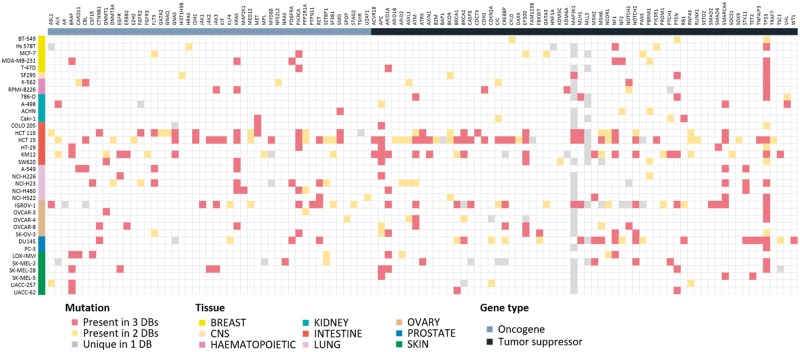
Mutational landscape of 125 driver genes defined by Vogelstein in 36 cell lines. Mutated genes were colored according to overlap in database sources. We omitted 16 genes with no mutations in our cell lines.

Mutation information is probably of uttermost interest. Because of unavailability of raw sequencing data, we collected and compared the final results from the source databases. Differences in sequencing and analysis details are expected to yield somewhat different mutation profiles. For 36 cell lines where all three resources report mutation data, we show the comparison plot in Figure 3 for 125 driver genes selected by Vogelstein and coworkers (13). About 214 (54.5%) mutations showed agreement in all three resources, but 110 (28.0%) mutations had agreement in only two resources. 69 (17.6%) mutations had resource-specific mutations. *BRAF*, *KRAS*, *PIK3CA*, *APC*, *ARID1A*, *NF1*, *PTEN*, *SMARCA4* and *TP53* genes were frequently mutated and their mutations were mostly common among the three resources. Many of *MAP3K1* and *MLL3* mutations were unique in the CCLE database with much higher frequency than expected, which raises some questions on the reliability of variant calling. Thus, users should be careful in assessing the significance of mutations reported from the original databases.

**Figure 4.**
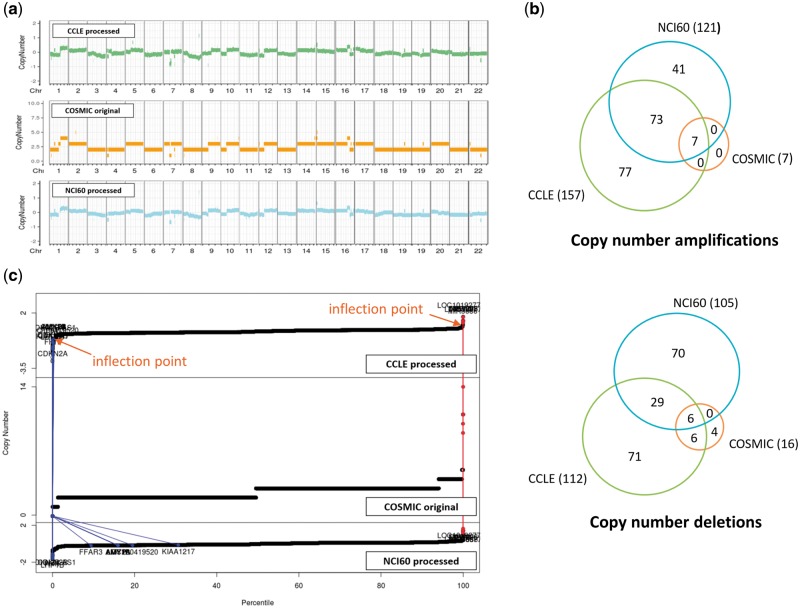
CNV data for NCI-H460 cell line from three resources. (**a**) CNV plots over chromosomes. COSMIC reports integer values, whereas our own processing of CCLE and NCI60 data sets gives real numbers. (**b**) Venn diagram of genes called as copy number amplifications and deletions. (**c**) Plots of CNV values for all genes in ascending order. Two inflection points were used to define copy number amplified (in red) and deleted (in blue) genes as described in Materials and methods section. We also show where the copy number altered genes by COSMIC appear in CCLE and NCI60 plots.

CNVs are even more complicated to compare. For two resources where raw data were available, we reprocessed the raw data and identified copy number aberrations using our own method as described in the Materials and methods section. For COSMIC which does not provide raw data, we just imported the processed CNV values (given in integer). CNV plots were produced for the NCI-H460 cell line, which is frequently used in lung cancer research (Figure 4a). Visual inspection indicated that CNV plots were in good agreement among the three resources, but calls for copy number amplifications and deletions yielded gene lists with partial overlaps as indicated in Figure 4b. We find that the COSMIC calls are biased toward copy number deletions (i.e. less number of copy number amplifications) in most cell lines. Note that both CCLE and NCI60 give a lot more aberrations with good overlaps, but COSMIC reports much less copy number aberrations probably because of different data processing methods and their policy favoring deletions over amplifications. Plots of CNV values of all genes in ascending order (Figure 4c) imply that CNV values are almost contiguous and it is not easy to select cut-off values to identify copy number amplifications and deletions.

### User interface and visualization

GEMiCCL supports cell line search, gene search, browsing with histological characteristics and complex queries based on the Boolean logic rules. Cell line search shows summary information, copy number, gene expression and mutation data displayed in separate tab pages.

**Figure 5.**
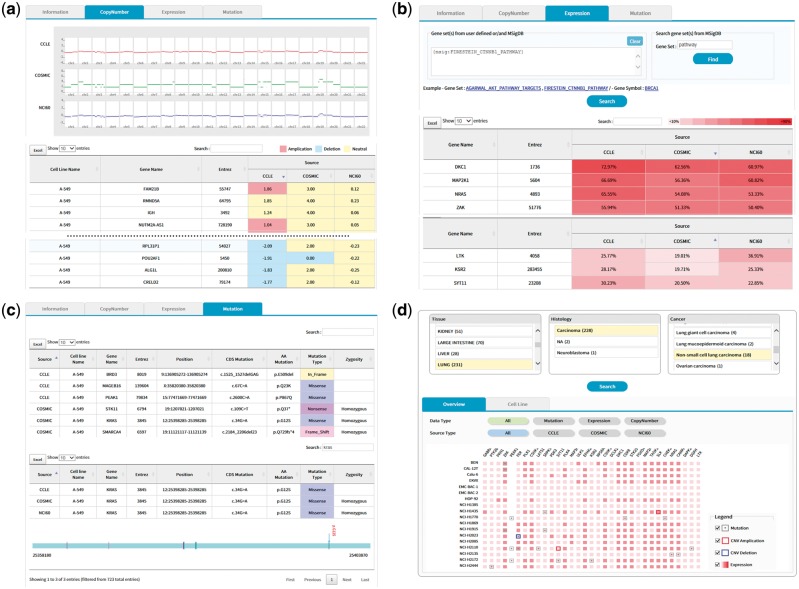
(a) CNV output from cell line search (A-549 cell line). Upper plots show the chromosomal CNV plots from three resources. Lower table shows the detailed copy number values where calls for amplification, deletion, and neutral were indicated in the background color. Note that the built-in sorting function of table utility was used to locate genes with copy number amplifications or deletions. **(b)** Gene expression output from cell line search (A-549 cell line). Users may specify a list of genes to be included for visualization in the upper panel. Alternatively, MSigDB can be used to define gene sets as shown in this example of CTNNB1 (β-catenin) pathway. Expression values normalized in the 0–100 percentile scale are shown in the table with background color indicating expression levels. Sorting or searching function of table utility can be used to locate genes of interest. **(c)** Mutation output from cell line search (A-549 cell line). The lower table was obtained by searching *KRAS* from the table search utility. A mouse click on any row shows the mutation plot on gene coordinate (bottom picture). Note that *KRAS* gene is shown in 3′←5′ direction. All genome coordinates are for hg19 human genome assembly. **(d)** Browsing of cancer cell lines. Selection on tissue-histology-cancer type works in successive way reflecting user’s choice dynamically. The lower panel is the summary of molecular characteristics over selected cell lines and genes. Again, gene list can be chosen via MSigDB as shown in (b). Presence of mutation is indicated in dots. Border color shows copy number amplifications and deletions. Intensity of background color indicates the gene expression level.

The copy number tab shows CNV plots from available data sources with a table of CNV values as shown in Figure 5. The CNV values can be sorted using the built-in sort function within the table to identify genes with copy number amplifications and deletions as colored in red and blue, respectively.

The expression tab shows a table of gene expression values again with sorting and searching functions (Figure 5b). Here the expression values are shown in percentiles among all genes in the cell line because a universal reference sample could not be defined. It is common for users to want to examine expression values only for a group of genes. We implemented a filtering scheme to allow users to input a list of interested genes. Alternatively, users may search the MSigDB (14) that contains 17 786 gene sets covering diverse curated pathways, gene ontology, targets of transcription factors and microRNAs and oncogenic and immunologic signatures. Figure 5b is an illustration of gene expression in the A-549 cell line for genes in the β-catenin pathway as defined by the MSigDB. This feature should facilitate users to concentrate on activities of genes or pathways of interest.

The mutation tab displays a collection of mutations in the selected cell line. Exome sequencing usually identifies hundreds or thousands of mutations, making it difficult to see the gene of interest. Again, users may use the built-in search function of the table to locate specific genes (Figure 5c). In an effort to help users determine the functional significance of each mutation, we implemented a simple JavaScript visualization tool indicating where the mutation and functional domains exist in the gene coordinate.

It is often the case that users have a gene of particular interest. Gene searches rather than cell line searches show CNV, expression and mutation information for the user-specified gene across many cell lines, in a similar fashion to the cell line search. The role of cell lines and genes is just interchanged in the output pages.

We also support a browsing function that combines tissue, histology and cancer types in a sequential order. Figure 5d shows the browsing result for 18 cell lines from non-small cell lung carcinoma. Each square in the overview/summary plot simultaneously indicates the presence of mutations, copy number alterations and expression levels for user-selected cell lines and genes. In this example, we added a filter for the β-catenin pathway genes in the MSigDB defined in the same way as in Figure 5b. Users may choose to see specific data types or resources using the checkbox options. The cell line tab provides a table that includes the number of copy number-altered genes, expressed genes and mutated genes for selected cell lines. Clicking each number opens a new window to show the gene list with more detailed information.

Lastly, we support a smart search scheme based on the combination of Boolean logic rules. Users may enter query terms in any combination of histological characteristics and gene names. We also support diverse types of query tags including CNV type, mutation, expression and DB sources. A search of cell lines from lung adenocarcinoma with BRAF mutation can be easily accomplished using this Boolean logic search. A query builder was implemented to help users compose complex queries in accordance with our query tags.

## Conclusion

Clinical and molecular characteristics are two most important factors in selecting cancer cell lines for functional or screening experiments. GEMiCCL is the most comprehensive database of cancer cell lines to date, covering gene expression, mutation and copy number information, integrated carefully from three representative resources of CCLE, COSMIC and NCI60. It also supports users to compare such properties from different resources objectively without batch effect. Furthermore, experimental scientists should be able to look up molecular characteristics easily with our user-friendly web interface and interactive features.
